# The effects of medicines availability and stock-outs on household’s utilization of healthcare services in Dodoma region, Tanzania

**DOI:** 10.1093/heapol/czz173

**Published:** 2020-01-13

**Authors:** August Kuwawenaruwa, Kaspar Wyss, Karin Wiedenmayer, Emmy Metta, Fabrizio Tediosi

**Affiliations:** 1 Ifakara Health Institute, Plot 463, Kiko Avenue Mikocheni, PO Box 78 373, Dar es Salaam, Tanzania; 2 Swiss Tropical and Public Health Institute (Swiss TPH), Basel, Switzerland; 3 University of Basel, Basel, Switzerland; 4 Swiss Centre for International Health (SCIH), Basel, Switzerland; 5 Health promotion and System Strengthening (HPSS), Dodoma, Tanzania; 6 School of Public Health and Social Sciences (SPHSS), Muhimbili University of Health and Allied Sciences (MUHAS), Dar es Salaam, Tanzania

**Keywords:** Medicines availability, healthcare utilization, Dodoma, Tanzania

## Abstract

Low- and middle-income countries have been undertaking health finance reforms to address shortages of medicines. However, data are lacking on how medicine availability and stock-outs influence access to health services in Tanzania. The current study assesses the effects of medicine availability and stock-outs on healthcare utilization in Dodoma region, Tanzania. We conducted a cross-sectional study that combined information from households and healthcare facility surveys. A total of 4 hospitals and 89 public primary health facilities were surveyed. The facility surveys included observation, record review over a 3-month period prior to survey date, and interviews with key staff. In addition, 1237 households within the health facility catchment areas were interviewed. Data from the facility survey were linked with data from the household survey. Descriptive analysis and multivariate logistic regressions models were used to assess the effects of medicine availability and stock-outs on utilization patterns and to identify additional household-level factors associated with health service utilization. Eighteen medicines were selected as ‘tracers’ to assess availability more generally, and these were continuously available in ∼70% of the time in facilities across all districts over 3 months of review. The main analysis showed that household’s healthcare utilization was positively and significantly associated with continuous availability of all essential medicines for the surveyed facilities [odds ratio (OR) 3.49, 95% confidence interval (CI) 1.02–12.04; *P* = 0.047]. Healthcare utilization was positively associated with household membership in the community health insurance funds (OR 1.97, 95% CI 1.23–3.17; *P* = 0.005) and exposure to healthcare education (OR 2.75, 95% CI 1.84–4.08; *P* = 0.000). These results highlight the importance of medicine availability in promoting access to health services in low-income settings. Effective planning and medicine supply management from national to health facility level is an important component of quality health services.

**Key Messages**

- The availability of quality medicines in the provision of healthcare service is an integral part of universal health coverage, shapes health service delivery as well as household healthcare utilization.
- Availability of most tracer medicines was relatively good, with frequent stock-outs of a few medicines and variation across level of care and across districts.
- Better forecasting of upcoming medicine needs and timely ordering at health facilities, along with the improved availability of medicines at the medical store department, could help prevent stock-outs and improve availability.

## Introduction

The availability of quality medicines in the provision of healthcare service is an integral part of universal health coverage (UHC; Prinja *et al.*, 2015). Evidence suggests that the availability of medicines is essential for healthcare service delivery (Obare *et al.*, 2014; Bigdeli *et al.*, 2015). In low- and middle-income countries (LMICs) the availability of essential medicines in public health facilities ranges from 17.9% to 87.1% (WHO, 2014). The proportion of total health expenditure spent on medicines in low-income countries ranges widely from 7.7% to 67.6% (Lu *et al.*, 2011). In addition, a limited proportion of funds for medicines is allocated to primary health facilities in these countries (Kusemererwa *et al.*, 2016). Coupled with frequent supply chain management problems (Cameron *et al.*, 2009; YALE, 2011), access to quality medicines is often limited, contributing to inequalities and deficits in access to health services and ultimately influencing health outcomes (Chen *et al.*, 2014; Masters *et al.*, 2014). The lack of medicines in health facilities fosters the use of over-the-counter medicines or products from unqualified sources, exposing consumers to the risks of using counterfeit or substandard products (Jia *et al.*, 2016). In addition, a large proportion of medicines is paid for out-of-pocket, potentially exposing households to financial hardship (Lu *et al.*, 2011; Prinja *et al.*, 2015; Luiza *et al.*, 2016).

Several LMICs have undertaken or are undertaking health finance reforms (Mtei *et al.*, 2007; Fenny *et al.*, 2018) aimed at increasing the availability of medicines at an affordable cost. The emphasis of some reforms has been on expansion of social health insurance schemes (Mcintyre *et al.*, 2013; Fenny *et al.*, 2018) and direct health facility financing (Kapologwe *et al.*, 2019) as a means of generating more revenue to purchase medicines and to protect citizens from the risk of catastrophic health expenditure (Saksena *et al.*, 2011; Kusi *et al.*, 2015; Ataguba and Ingabire, 2016). Furthermore, other initiatives undertaken include the strengthening of public–private partnerships as a means of ensuring healthcare facilities have sufficient medicines to carter for the population needs throughout the year (WHO, 2000; Rutta *et al.*, 2015; Embrey *et al.*, 2016; USAID, 2016).

The continuous availability of essential medicines within primary healthcare facilities plays an important role in promoting access to and utilization of health services. On the other hand, frequent stock-outs of medicines have been shown to influence healthcare utilization (Mwabu *et al.*, 1993; Anselmi *et al.*, 2015). In Uganda, the continued absence of medicines in public health facilities was found to influence healthcare utilization and individual decisions to consult health service providers (Shaikh and Hatcher, 2005; Musoke *et al.*, 2014). The availability of medicines positively affects patient trust in healthcare providers (Shan *et al.*, 2016), while medicine stock-outs in facilities foster distrust in healthcare providers and contributes to low utilization of the formal healthcare system (Mkoka *et al.*, 2014). Trust in healthcare providers is important; it shapes household healthcare utilization patterns, it influences medication adherence (Brennan *et al.*, 2013) and it fosters communication with service providers (Al-Mandhary *et al.*, 2007). A number of other factors have also been found to influence household healthcare-seeking behaviour. In rural areas, long distances to reach a formal healthcare provider results in the underuse of health services (Tran *et al.*, 2016). The availability of sufficient transport systems and close geographic proximity to healthcare facilities positively influences healthcare service utilization (Shaikh and Hatcher, 2005; Feikin *et al.*, 2009). In addition, affordability of healthcare services influences household’s healthcare-seeking behaviour; evidence suggest that health insurance increases the probability of households seeking care (Saksena *et al.*, 2011; Chomi *et al.*, 2014; Ahmed *et al.*, 2018; Atnafu *et al.*, 2018) and protects them against impoverishment from out-of-pocket expenditures (Saksena *et al.*, 2011; Spaan *et al.*, 2012).

To our knowledge, most studies assessing how medicine availability influences healthcare utilization in Tanzania have focused on specific diseases such as diarrhoea, fever/malaria, tuberculosis, chronic diseases and acute respiratory infection (Mikkelsen-Lopez *et al.*, 2013; Kante *et al.*, 2015; Nnko *et al.*, 2015; Senkoro *et al.*, 2015); on special vulnerable populations such as people who inject drugs; or on the influence of health insurance systems overall (Chomi *et al.*, 2014; Mlunde *et al.*, 2016). However, no study investigated how medicine availability and stock-outs influence healthcare utilization in the general population by integrating information from the healthcare facility and households. This study assesses the effects of medicine availability and stock-outs on healthcare utilization in Dodoma region, Tanzania by combining data of households and health facilities survey.

### Study setting

Tanzania has a decentralized health system which gives district councils authority to manage available resources district healthcare facilities. The central government, in turn, allocates funds to the medical store department (MSD), the main supplier of medicines, medical equipment and medical supplies to public healthcare facilities. However, several barriers challenge MSD’s effectiveness in supplying medicines to the health facilities, including inadequate funds for medicines, delays in disbursement of allocated funds, inaccurate forecasting of medicines at the facility and national level, thefts, stock-outs at the national MSD warehouse and ineffective systems for fulfilling back-ordered items (YALE, 2011). Nevertheless, health facilities and districts have funds available that are earmarked for purchasing supplementary medicines from the private sector when MSD is out-of-stock (HPSS, 2014). Funding for complementary medicines and supplies is closely linked with fiscal decentralization of public financial management and the community health fund (CHF ‘iliyoboreshwa’) system. These medicines are paid from the regular sources of complementary funds available such as those of the CHF, of the national health insurance fund (NHIF), of user fees, and the basket funds provided by the government, the donors and the private sector. However, the availability of medicines within the public sector in district-level facilities tends to be insufficient, in turn, affecting the quality of services. A survey conducted in 2012 in Dodoma region reported a stock-out rate of 46% and an order fulfilment rate of 59% from MSD (HPSS, 2011). The purchase of supplementary medicines has been reported to be fragmented, unco-ordinated, inefficient and lacking transparency (HPSS, 2014). In 2014, regional authority and district councils started implementing a complementary pharmaceutical supply system funded by Swiss Agency for Development and Cooperation through the Health Promotion and Systems Strengthening (HPSS) project known as Jazia Prime Vendor system (Jazia PVS). The aim of Jazia PVS is to improve the availability of medicines in the Dodoma region by complementing MSD supply. Jazia PVS is a unique public–private partnership between the health authorities of the Dodoma region and a private supplier (HPSS, 2014). The Jazia PVS consolidates and pools orders for supplementary medicines from all public healthcare facilities at the district level and purchases from one contracted supplier, the Prime Vendor. Medicines are paid for using the funds collected through national insurance schemes (CHF and NHIF), user fees and basket funds (Mushi, 2014). Jazia PVS was designed to address shortages of medicines in primary-level public health facilities by pooling the limited resources available from districts councils. Healthcare decision-makers require information on the effectiveness of the Jazia PVS, including the effect on medicines availability and stock-outs and on household healthcare utilization.

This study was carried out in six district councils in the Dodoma region in Tanzania where the Jazia PVS was implemented: Kondoa, Kongwa, Dodoma city council, Bahi, Mpwapwa and Chemba. Table 1 presents information about the included districts in Dodoma region. The region has a population of 2 083 588. Of the six district councils, Dodoma municipal has the largest population (410 956) whereas Bahi district council has the smallest population (221 645). Bahi has the largest average number of primary healthcare facilities per 10 000 population (1.8), followed by Chemba and Kongwa district councils (1.4) and Dodoma city council has the fewest (0.8).

**Table 1.** Districts council basic information

District council variable	Kondoa	Kongwa	Dodoma city	Bahi	Mpwapwa	Chemba
Population^a^	269 704	309 973	410 956	221 645	305 056	235 711
Area coverage (km^2^)	5921	4041	2576	5948	7479	7289
Number of public health centres^b^	2	4	7	6	2	4
Number of public dispensaries^b^	27	40	27	35	39	30
Number of private health facilities^b^	11	8	29	2	5	4
Number of primary care facilities per 10 000 population	1.1	1.4	0.8	1.8	1.3	1.4
Number of primary health facilities surveyed						
Hospital	1	1	1	0	1	0
Health centres	2	1	1	3	1	3
Dispensaries	6	16	13	12	18	13
Total staffing in the surveyed health centre						
Clinical cadre^c^	3	2	2	9	2	2
Nurse cadre^d^	14	4	0	39	4	4
Pharmacists cadre^e^	0	0	0	1	0	0
Total staffing in the surveyed dispensary clinical cadre	1	7	8	4	5	3
Nurse cadre	15	18	29	45	27	22
Pharmacists cadre	0	0	0	0	0	0
Household interviews						
Household selected (*n* = 1264)	194	223	296	160	221	170
Household interviewed (*n* = 1237)	195	220	281	168	201	172
Household response rate (%) (98.5)^f^	100.5	98.7	94.9	105	91.0	101.2

a NBS, Tanzania National Bureau of Statistics; Population and Housing Census 2013.

b http://hfrportal.ehealth.go.tz/ (accessed 15 January 2018; only operating facilities).

c Composed of Medical Doctor (MD), Assistant Medical Officer (AMO) and Clinical Officer (CO).

d Composed of Medical Attendant (Nurse Assistant), Nurse Midwife and Nurse Officer.

e Composed of Pharmacist, Pharmaceutical Assistant and Pharmaceutical Technician.

f Variation of household response rates by district was due to the fact that some of sampled iCHF households members have permanently/temporarily migrated out of the sampled villages as it was a harvesting time and some villages had changed their administrative boundaries hence the names of households do not appear in the sampled villages, therefore, there was a need to sample extra households.

## Methods

### Study design

Two cross-sectional surveys were conducted in May 2017 in Dodoma region: (1) a household survey and (2) a healthcare facility survey. The two surveys covered the same areas and were then combined together to assess the effects of medicines availability and stock-outs on household healthcare utilization.

### Health facility survey

The sample size for the healthcare facilities was 50% of all government health facilities (267) covered by the Health Promotion and System Strengthening programme in Dodoma region. The health facilities were stratified into three categories, namely hospitals, health centres and dispensaries. A probability proportional to size sampling design was utilized, whereby the number of health facilities selected was adjusted based on the number of healthcare facilities in the district. Thus districts with larger numbers of health facilities had a greater number of health facilities included in the sample. A total of 4 hospitals and 89 public primary healthcare facilities (11 health centres and 78 dispensaries) were randomly selected and surveyed in May 2017 across the seven districts. Surveys included observation, record review and interview with key staff at each health facility selected. Healthcare facility staff was interviewed to collect data on medicine availability, frequency and duration of medication stock-outs, reasons for stock-outs and facility staffing levels (Supplementary material S2: Sample of health facility survey tool). The survey addressed the previous 3-month period of February to April 2017.

The availability of 18 tracer medicines was examined from existing health facility records (Supplementary Table S1). The 18 tracer medicines were selected to align with the medications targeted by the HPSS-Jazia PVS. A pharmacist and an enumerator verified the availability and stock-outs of medications using a review of facility records from the previous 3 months (90 days) prior to the survey. The average number of days a facility had experienced stock-outs for each of 18 medicines was recorded (Supplementary Table S2). We categorized health facilities as those with and without any stock-outs over the observation period of 3 months prior to the survey date and this variable was included in the final regression model.

### Household survey

A multi-stage sampling approach was used in the selection of wards and villages from the councils. In the first stage, a list of wards was obtained and three wards from each district were randomly selected. The second stage of selection involved the random selection of two villages from each ward. In total, 42 villages were chosen across the district councils. The sample size was obtained by adopting a formula from Cochran with consideration of households who had enrolled in CHF and those who are not enrolled (Cochran, 1977). A random sample of 1237 households was interviewed from the villages. At the village level, households were categorized into two categories, the first group consisted of those who were previously enrolled in the CHF ‘iliyoboreshwa’ scheme (415 households) that were randomly selected for interview from the Insurance Management Information System database. While the second group were non-CHF members (822 households) that were randomly selected from a list of all households in the village, obtained from a village chairperson. At each household, the head of the household or his/her representative was interviewed to collect information on household demographic and economic characteristics, healthcare access and utilization. Demographic and economic characteristics included ownership of assets, household income and expenditure and health insurance status. Recent healthcare utilization, illness episodes and health problems, reasons for not consulting health services; waiting times at healthcare facilities where care was sought, distance from the closest healthcare facility, trust to healthcare providers and exposure to health education were also assessed. Potential respondents aged 18 years and above were eligible to participate. In this study, health education has been conceptualized as one of the strategies of health promotion intended to raise community awareness of relevant health issues and enhance knowledge in improving health such as preventing illness and seeking timely and appropriate health assistance.

### Data collection and management

A team of six experienced supervisors, 5 district pharmacists and 21 enumerators were recruited for field data collection. In each district, a pharmacist and one enumerator conducted the health facility survey. Four enumerators implemented the household’s survey. All supervisors, pharmacists and enumerators together with research scientists underwent a 3 days training session. Health facility and household survey tools were pre-tested villages in Dodoma rural district council that were included in the study sample. Open Data Kit technologies on Android mobile devices were used for data collection and management in both surveys. Data from both surveys were exported and analysed in STATA version 13.0. The household and healthcare facility response rate across all the district councils was 98.5% and 100%, respectively.

Data from facility surveys were linked with data from household surveys conducted in the same geographical location. To this end, we first used the household information on place of residence (such as village and ward/street) to match households with facilities in the same village or area. Secondly, we then matched the two surveys using global positioning system co-ordinates of both health facilities and households’ village to visualize the spatial distribution of households and health facilities using the ArcGIS software v10.5 (ESRI, Redland, CA, USA). The shapefiles of Dodoma region were obtained from the National Bureau of Statistics (http://www.nbs.go.tz/) and geo-processing was used to dissolve to the district level. The results for the second stage are presented in Supplementary Figure S1: Map of Dodoma showing the distribution of healthcare facilities and households surveyed. A total of 577 households out of 1237 (47%) surveyed households were successfully linked across six out of the seven district councils. We could not include one district, Chamwino, in the study due to the fact that none of the 20 facilities surveyed was in the catchment area of the households surveyed (232).

### Analysis

#### Descriptive statistics

Descriptive statistics were generated for the health facility and household survey data. We computed frequencies and percentages of reported medicine availability/stock-outs considering facilities with and without any stock-out of medicines within the observation period of 90 days (3 months). The mean value of medicines availability in the surveyed facilities was 0.73 with the minimum–maximum value of (0.22–1.00; Supplementary Table S3).

Descriptive statistics were used to summarize household economic and demographic characteristics and healthcare utilization. We then used ‘*t*-tests’ to assess whether the difference in proportions between districts for each variable was statistically significant. The descriptive statistics informed the variables (covariates) included in the multivariate logistic regression model, to assess the effects of medicines availability and stock-outs on healthcare utilization.

#### Empirical strategy

A ‘Pearson’s correlation’ analysis was used to examine the strength and direction of the linear relationship between facilities without any stock-outs and household use of public healthcare facilities. We hypothesized that household healthcare utilization would be affected by a continuous availability of medicine and stock-outs. Other variables which could affect healthcare utilization included sociodemographic variables, CHF insurance coverage, level of trust in facility staff, receiving healthcare education, waiting time at the health facility, distance to the facility, chronic illness in at least one household member and household income (Supplementary Table S5). Backward elimination was used to arrive at the final model, a technique in which variables with the highest *P*-values were eliminated one by one, conditional on the *P*-value being bigger than some pre-determined level (*P* > 0.60). Furthermore, the models were subjected to a diagnostic test to ensure the model was correctly specified; we used the link test for model specification (Long and Freese, 2006). The regression analysis has been clustered at the facility level, relaxing the assumption of independence (Cameron and Miller, 2015).

We created a household wealth index including indicators relating to housing characteristics (water source, toilet type, nature of the flooring, nature of roof) and assets (electricity, radio, TV, mobile phone, car, refrigerator, bicycle) using polychoric principal component analysis (Vyas and Kumaranayake, 2006). The constructed wealth index was used as a proxy measure of the household living standard; households were ranked according to the wealth index score and generated wealth quintiles of each household, five equally sized groups. Sampled households were classified according to the five wealth quintiles.

## Results

### Descriptive statistics

#### Availability of medicines in healthcare facilities

Table 2 presents results on the availability of 18 tracer medicines in the sampled facilities along with the mean days of medicine stock-outs in the 3 months prior to the survey (Supplementary Table S2). Availability of artemether/lumefantrine (ALU) was generally high in all facilities in all districts above 85.7%. Availability of amoxicillin caps or cotrimoxazole tabs was above 70.0% in five districts, except Chemba district where availability was 57.8%. Availability of Amoxicillin syrup and cotrimoxazole suspension in all facilities in all districts was below 65%, with 73.3%, 70.6% and 68.4% of facilities in Kongwa, Bahi and Mpwapwa, respectively, experiencing stock-outs for >14 days. We found that availability of Ceftriaxone 1 g injection/250 g injection in all facilities in all districts was above 85.0% in Chemba and Kondoa districts.

**Table 2.** Availability of medicine for the last 3 months prior to the date of the survey

District name (*n* = number of facilities), no stock-out was observed for 90 days (%)	Kondoa (*n* = 8)	Kongwa (*n* = 17)	Dodoma city (*n* = 14)	Bahi (*n* = 15)	Mpwapwa (*n* = 19)	Chemba (*n* = 16)	Total (*n* = 89)
ALU oral^a^	100.0	100.0	85.7	100.0	94.7	100.0	96.6
Quinine injection or artesunate injection^b^	62.5	70.6	64.3	93.3	89.5	93.7	80.9
Amoxicillin caps or cotrimoxazole tabs^a^	87.5	70.6	85.7	93.3	57.8	93.1	79.8
Amoxicillin syrup or cotrimoxazole suspension	62.5	29.4	42.9	20.0	31.6	56.3	38.2
Benzyl penicillin 5 MU injection^a^	87.5	41.2	35.7	80.0	73.7	93.6	67.4
Ceftriaxone 1 g injection/250 g injection^c^	87.5	52.9	64.3	53.3	52.6	93.6	65.2
Mebendazole or albendazole tabs^a^	87.5	64.7	78.6	86.7	52.6	81.3	73.0
Griseofulvin oral or clotrimoxazole cream^c^	87.5	17.6	64.3	60.0	73.7	75.0	60.7
Metronidazole tabs^a^	100.0	76.5	78.6	100.0	63.2	100.0	84.3
ORS sachet^a^	87.5	64.7	64.3	73.3	57.8	93.7	71.9
Paracetamol 500 mg tabs^c^	100.0	64.7	71.4	33.3	47.4	100.0	66.3
Medroxyprogesterone acetate (depo) injection^a^	100.0	94.1	100.0	93.3	73.7	100.0	92.1
Oxytocin injection^a^	100.0	100.0	85.7	100.0	100.0	100.0	97.8
Ferrous salt and folic acid^c^	50.0	11.8	50.0	26.7	31.6	0.0	25.8
Vaccine, e.g. DTP vaccine^a^	100.0	100.0	78.6	93.3	89.4	100.0	93.3
Ophthalmologic drops or cream^a^	87.5	58.8	71.4	53.3	84.2	100.0	75.3
Dextrose 5% or DNS or Ringer solution^c^	87.5	64.7	64.3	93.3	42.1	100.0	73.0
Adrenaline injection^c^	87.5	52.9	57.1	80.0	100.0	93.6	78.6

a Significance at 5% level.

b Significance at 10% level.

c Significance at 1% level.

DNS, Dextrose normal saline; ORS, Oral rehydration salts.

Availability of paracetamol 500 mg tabs was generally high in all facilities in Kondoa and Chemba reaching 100.0%. However, it was lower in Bahi and Mpwapwa, below 50.0%, with several facilities reporting stock-out for >14 days (53.3% and 31.6%, respectively). Availability of oxytocin injection was generally high in all facilities in all districts above 85.6%. A 100% availability of diphtheria, tetanus and pertussis (DTP) vaccine was reported in Kondoa, Kongwa and Chemba district councils.

Availability of ferrous salt and folic acid was below 52.0% across all the facilities in all the districts, and most facilities reported stock-outs of >14 days. About 41.2% and 35.7% of facilities in Kongwa and Dodoma city, respectively, experienced stock-out of adrenaline injection that lasted >14 days.

Availability of individual medicines by facility level over the observed period of 3 months is presented in Supplementary Table S1. The availability of most medications varied substantially across facility levels. All health centres had 100.0% availability of ALU, whereas 96.2% of the dispensaries had ALU. Mebendazole was available in 72.7% of health centres and 73.1% of dispensaries. All health centres had a 63.5% availability of paracetamol, compared with 66.7% of dispensaries. Only 28.2% of dispensaries and 9.1% of health centre had ferrous salt in stock.

**Figure 1.**
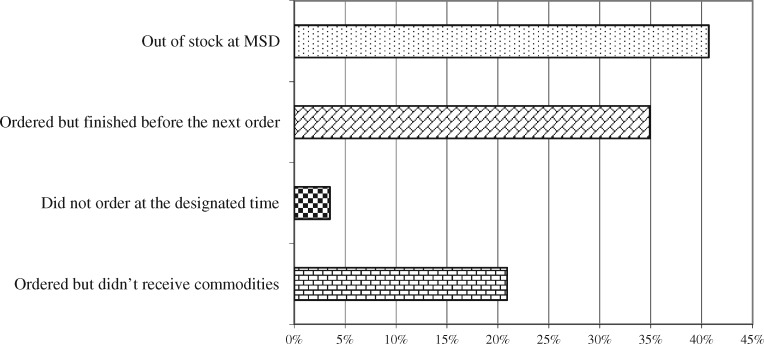
Reasons for the out of stock for the past 3 months.

Out of 89 healthcare facility surveyed the most commonly reported reasons for the medicines stock-out were lack of availability of medication at MSD (40.7%), use of all stocked medicines before the next order arrived (34.9%), failure to receive medicines that had been ordered (20.9%) and failure of facility to send orders at designated time (3.5%; Figure 1).

#### Household’s demographic and socioeconomic characteristics

Table 3 presents information on the participant’s demographic and socioeconomic characteristics. The majority of the surveyed households were male-headed (68.6%). The average age of respondents was 49.6 years [standard deviation (SD): 16.2]. Most heads of the households were aged between 46 and 64 years (31.3%), or between 36 and 45 years (26.2%). Few households were headed by someone below 25 years of age (5.2%). Most of the heads of households (68.3%) had attended primary school up to grade five while few (5.7%), had secondary education and above, and 26.0% had not attended formal education. About half (48.9%) heads of households were farmers, 24.3% were not employed and very few (2.4%) were employed in a formal sector position. The majority (58.4%) of respondents were not married. About 75.6% of respondents reported their health as ‘good’ while few (1.9%) reported ‘bad’ health status. The average household size across all the district councils was 4.5 people (SD: 1.9; Table 3).

**Table 3.** Demographic and socioeconomic characteristics of the respondents included in the analysis

Variable	Kondoa (*n* = 79)	Kongwa (*n* = 24)	Dodoma city (*n* = 200)	Bahi (*n* = 65)	Mpwapwa (*n* = 129)	Chemba (*n* = 80)	Total (*n* = 577)
Gender of head of household, *n* (%)							
Male	45 (56.0)	8 (33.3)	123 (61.5)	53 (81.5)	93 (72.1)	66 (82.5)	396 (68.6)
Female	34 (43.9)	16 (66.7)	77 (38.5)	12 (18.5)	36 (27.9)	14 (17.5)	181 (31.4)
Age categories of head of household							
≤25, *n* (%)	0 (0.0)	3 (12.5)	13 (6.5)	5 (7.7)	5 (3.9)	4 (5.0)	30 (5.2)
26–35, *n* (%)	5 (6.3)	3 (12.5)	39 (19.5)	14 (21.5)	23 (17.8)	15 (18.7)	99 (17.2)
36–45, *n* (%)	17 (21.5)	6 (25.0)	47 (23.5)	18 (27.7)	40 (31.0)	23 (28.7)	151 (26.2)
46–64, *n* (%)	27 (34.2)	12 (50.0)	58 (29.0)	21 (32.3)	34 (26.4)	29 (26.3)	181 (31.4)
≥65, *n* (%)	30 (38.0)	0 (0.0)	43 (21.5)	7 (10.8)	27 (20.9)	9 (11.3)	116 (20.0)
Mean (years) (SD)	60 (17.9)	45 (10.4)	49 (16.2)	45 (14.0)	48 (115.8)	46 (13.3)	49.6 (16.2)
Education level of head of household, *n* (%)							
No education	35 (44.3)	5 (20.8)	42 (21.0)	24 (36.9)	32 (24.8)	12 (15.0)	150 (26.0)
Primary up to grade five	43 (54.4)	18 (75.0)	133 (66.5)	41 (63.1)	95 (73.6)	64 (80.0)	394 (68.3)
Secondary and above	1 (1.3)	1 (4.2)	25 (12.5)	0 (0.0)	2 (1.6)	4 (5.0)	33 (5.7)
Occupation of head of household, *n* (%)							
Formal employed	0 (0.0)	0 (0.0)	7 (3.5)	1 (1.5)	3 (2.3)	3 (3.7)	14 (2.4)
Farmer	39 (49.4)	21 (87.5)	39 (19.5)	57 (87.7)	73 (56.6)	53 (66.3)	282 (48.9)
Self-business	8 (10.1)	0 (0.0)	94 (47.0)	1 (1.5)	27 (20.9)	11 (13.7)	141 (24.4)
Not employed	32 (40.5)	3 (12.5)	60 (30.0)	6 (9.2)	26 (20.2)	13 (16.3)	140 (24.3)
Marital status, *n* (%)							
Married	57 (72.2)	13 (54.2)	74 (37.0)	39 (60.0)	43 (33.3)	51 (63.7)	240 (41.6)
Not married	22 (27.8)	11 (45.8)	126 (63.0)	26 (40.0)	86 (66.7)	29 (36.3)	337 (58.4)
Health status of head of household, *n* (%)							
Good	53 (67.1)	19 (79.2)	143 (71.5)	53 (81.5)	105 (79.1)	66 (82.5)	436 (75.6)
Average	25 (31.6)	5 (20.8)	50 (25.0)	11 (16.9)	25 (19.4)	14 (17.5)	130 (22.5)
Bad	1 (1.3)	0 (0.0)	7 (3.5)	1 (1.5)	2 (1.5)	0 (0.0)	11 (1.9)
Number of people in the household							
≤2, *n* (%)	17 (21.5)	1 (4.2)	33 (16.5)	4 (6.2)	17 (13.2)	14 (17.5)	86 (14.9)
3–4, *n* (%)	30 (38.0)	6 (25.0)	75 (37.5)	18 (27.7)	66 (51.2)	30 (37.5)	225 (38.9)
5–6, *n* (%)	23 (29.1)	13 (54.2)	55 (27.5)	32 (49.2)	31 (24.0)	27 (33.7)	181 (31.4)
≥7, *n* (%)	9 (11.4)	4 (16.6)	37 (18.5)	11 (16.9)	15 (11.6)	9 (11.3)	85 (14.7)
Average house hold size (SD)	4.2 (1.8)	5.0 (1.4)	4.6 (2.0)	5.0 (1.7)	4.2 (1.7)	4.4 (2.1)	4.5 (1.9)
CHF insurance status, *n* (%)							
CHF insured	72 (91.1)	9 (37.5)	41 (20.5)	8 (12.3)	25 (19.4)	2 (2.5)	157 (27.2)
Not insured	7 (8.9)	15 (62.5)	159 (79.5)	57 (87.7)	104 (80.6)	78 (97.5)	420 (72.8)
Social economic status (%), *n* (%)							
S1 (poorest)	35 (44.3)	1 (4.2)	48 (24.0)	5 (7.7)	24 (18.6)	12 (15.0)	125 (21.7)
S2	13 (16.5)	7 (29.2)	21 (10.5)	25 (38.5)	7 (5.4)	22 (27.5)	95 (16.5)
S3	10 (12.7)	9 (37.5)	30 (15.0)	24 (36.9)	44 (34.1)	31 (38.7)	148 (25.6)
S4	9 (11.4)	5 (20.8)	38 (19.0)	7 (10.8)	34 (26.4)	8 (10.0)	101 (17.5)
S5 (non-poor)	12 (15.1)	2 (8.3)	63 (31.5)	4 (6.2)	20 (15.5)	7 (8.8)	108 (18.7)

#### Household healthcare utilization

Among the households which were successfully linked with the health facility providing services in their region, 255 (44.2%) reported an illness episode of a household member in the last 3 months prior to the survey. The reported causes of illness were chest and related diseases (20.3%), malaria (18.0%) and typhoid and stomach-related diseases (12.9%). Out of 255 households, ∼7.8% reported a member with non-communicable diseases (NCDs—point prevalence) such as cancer, hypertension and diabetes (7.8%), fever (5.5%), illness related to eyes and ears (3.9%), urinary tract infection (3.1%), while the health problem could not be specified for 12.2% (Table 4).

**Table 4.** Healthcare utilization

Illness episode last 3 months	Kondoa (*n* = 79), *n* (%)	Kongwa (*n* = 24), *n*(%)	Dodoma city (*n* = 200), *n* (%)	Bahi (*n* = 65), *n* (%)	Mpwapwa (*n* = 129), *n* (%)	Chemba (*n* = 80), *n* (%)	Total (*n* = 577), *n* (%)
Household reported any illness case	44 (55.7)	10 (41.7)	78 (39.0)	33 (50.8)	55 (42.6)	35 (43.7)	255 (44.2)
Type of Illness episode reported							
Malaria	10 (22.7)	3 (30.0)	9 (11.5)	3 (9.1)	12 (21.8)	9 (25.7)	46 (18.0)
Urinary tract infection	1 (2.3)	1 (10.0)	3 (3.8)	0 (0.0)	1 (1.8)	2 (5.7)	8 (3.1)
Eyes and ears	3 (6.8)	0 (0.0)	4 (5.1)	1 (3.0)	2 (3.6)	0 (0.0)	10 (3.9)
Fever	5 (11.4)	0 (0.0)	3 (3.8)	3 (9.1)	1 (1.8)	2 (5.7)	14 (5.5)
Typhoid and stomach-related diseases	6 (13.6)	1 (10.0)	14 (17.9)	4 (12.1)	2 (3.6)	6 (17.1)	33 (12.9)
Chest-related diseases	13 (29.6)	3 (30.0)	12 (15.4)	4 (12.1)	10 (18.2)	11 (31.4)	53 (20.3)
Cancer, pressure and diabetes (NCDs)	1 (2.3)	0 (0.0)	15 (19.2)	0 (0.0)	3 (5.5)	1 (2.8)	20 (7.8)
Others	5 (11.4)	2 (0.0)	17 (21.8)	2 (6.1)	10 (18.2)	4 (11.4)	40 (15.7)
No information on the type of illness	0 (0.0)	0 (0.0)	1 (1.3)	16 (48.5)	14 (25.5)	0 (0.0)	31 (12.2)
Household sought help	36 (81.8)	10 (100)	70 (89.7)	15 (45.5)	37 (67.3)	32 (91.4)	200 (78.4)
Where did she/he go for treatment							
Public dispensary or health centre	30 (81.1)	8 (80.0)	16 (22.9)	9 (60.0)	22 (61.1)	19 (59.4)	104 (52.0)
Private doctor/clinic	0 (0.0)	0 (0.0)	7 (10.0)	0 (0.0)	5 (13.9)	0 (0.0)	12 (6.0)
Public hospital	2 (5.41)	0 (0.0)	20 (28.6)	3 (20.0)	5 (13.9)	4 (12.5)	34 (17.0)
NGO or trust hospital/clinic	0 (0.0)	0 (0.0)	1 (1.4)	0 (0.0)	0 (0.0)	0 (0.0)	1 (0.5)
Private hospital	0 (0.0)	0 (0.0)	10 (14.3)	1 (6.7)	0 (0.0)	1 (3.1)	12 (6.0)
Traditional healer	0 (0.0)	0 (0.0)	1 (1.4)	1 (6.7)	3 (8.3)	0 (0.0)	5 (2.5)
Pharmacy/drugstore	5 (13.5)	2 (20.0)	14 (20.0)	1 (6.7)	0 (0.0)	6 (18.7)	28 (14.0)
Home treatment	0 (0.0)	0 (0.0)	0 (0.0)	0 (0.0)	0 (0.0)	0 (0.0)	0 (0.0)
Local doctor	0 (0.0)	0 (0.0)	1 (1.4)	0 (0.0)	1 (2.8)	2 (6.3)	4 (2.0)
The reason that the sufferer not sought care							
Ailment not considered serious	1 (14.3)	0 (0.0)	0 (0.0)	1 (5.6)	1 (5.6)	0 (0.0)	3 (5.5)
Expected to become better without treatment	0 (0.0)	0 (0.0)	0 (0.0)	0 (0.0)	1 (5.6)	0 (0.0)	1 (1.8)
No drugs available in the area	1 (14.3)	0 (0.0)	0 (0.0)	0 (0.0)	2 (11.1)	0 (0.0)	3 (5.5)
Did not believe it would help	0 (0.0)	0 (0.0)	0 (0.0)	0 (0.0)	0 (0.0)	0 (0.0)	0 (0.0)
Consultation and drugs too expensive	0 (0.0)	0 (0.0)	3 (37.5)	0 (0.0)	0 (0.0)	0 (0.0)	3 (5.5)
Took self-treatment	4 (57.1)	0 (0.0)	4 (50.0)	1 (5.6)	1 (5.6)	3 (100)	13 (23.6)
No reason given	2 (14.3)	0 (0.0)	1 (12.5)	16 (88.8)	13 (72.2)	0 (0.0)	32 (58.2)

NGO, Non-governmental organization.

Of the 255 who reported illness, 200 (78.4%) sought care from a healthcare provider. About 52.0% of them sought healthcare from public dispensary or health centre, whereas 17.0% from public hospital, 14.0% sought care from pharmacy/drugstore, 6.0% from private hospital, 6.0% sought care from private doctor/clinic, 2.0% sought care from local doctor and 2% sought care from traditional healer (Table 4).

The reasons given for not seeking care were the health problem was not considered serious (5.5%); no drugs were available in the area (5.5%); participant perceived that consultation and drugs were too expensive (5.5%); participant expected to recover without treatment (1.8%); individual had knowledge on how to deal with the health problem and took self-treatment (23.6); and the remaining 58.2% did not report a reason for not seeking care with illness (Table 4).

#### Multivariate logistic regression

The link test showed that the model was correctly specified (Supplementary Table S6). Table 5 presents a multivariate logistic regression analysis on the effects of medicine availability on the household’s healthcare utilization. Results show that households with self-reported good health status were two times [odds ratio (OR) 1.80, 95% confidence interval (CI) 1.06–3.05; *P* = 0.029] as likely to seek care from formal healthcare providers compared with respondents that reported bad health status. Households that had received health education interventions were >2.7 times as likely (OR 2.75, 95% CI 1.84–4.08; *P* = 0.000) to seek healthcare services as were those who had not received health education. Results on pairwise correlation matrix showed a positive and significant association between the healthcare utilization and with facilities without any stock-outs (0.197) together with less waiting time at the facility (0.136), while a negative association was observed with minutes taken to reach at the healthcare facility when accessing healthcare services (−0.040) (Supplementary Table S4). Regression results showed that households that reported <60 min of wait time during the previous healthcare facility visit were more likely to have sought care than those that waited >60 min (OR 2.02, 95% CI 0.75–5.44; *P* = 0.167). In addition, households that were member of a community health insurance fund (CHF) were two times as likely to seek care from a formal provider than those not registered (OR 1.97, 95% CI 1.23–3.17; *P* = 0.000).

**Table 5.** Multivariate logistic regression on the effects of medicines availability and stock-outs healthcare utilization

Variable, odds ratio (confidence interval)	Univariate analysis (255)		Multivariate analysis (255)	
	OR (95% CI)	*P*-value	OR (95% CI)	*P*-value
Age of respondents	0.998 (0.98–1.02)	0.869	0.992 (0.97–1.01)	0.441
Household head being male	1.185 (0.69–2.03)	0.538	1.365 (0.43–4.25)	0.591
Household head being married	0.629 (0.33–1.20)	0.161	0.532 (0.24–1.15)	0.107
Household self-reported good health status^a^	1.737 (1.08–2.78)	0.021	1.801 (1.06–3.05)	0.029
Household being a CHF membership^a^	2.212 (1.11–4.42)	0.024	1.974 (1.23–3.17)	0.005
Level of trust to facility staffs being great	1.359 (0.82–2.25)	0.234	1.307 (0.76–2.24)	0.338
Household head received healthcare education^b^	1.912 (1.23–2.98)	0.004	2.745 (1.84–4.08)	0.000
Waiting time at the health facility <60 min	1.783 (0.85–3.74)	0.126	2.015 (0.75–5.44)	0.167
Distance to the facility <5 km	1.107 (0.56–2.19)	0.769	1.624 (0.74–3.54)	0.225
Minutes to the closest facility	0.998 (0.99–1.00)	0.558		
Household with at least one person with chronic illness	0.856 (0.60–1.22)	0.397	0.872 (0.54–1.40)	0.575
Facilities without any stock-outs for the past 3 months^a^	4.869 (1.75–20.18)	0.029	3.496 (1.02–12.04)	0.047
Household size	0.994 (0.89–1.11)	0.909	0.986 (0.77–1.26)	0.908
Wealth index value (proxy of income)	0.868 (0.76–0.99)	0.043	0.908 (0.80–1.02)	0.116
Total number of staffs	1.089 (0.79–1.49)	0.596		
TASAF beneficiary	1.856 (0.79–4.35)	0.154	0.991 (0.50–1.95)	0.978
Waiver/exemption of any household member	1.117 (0.56–2.21)	0.751	1.056 (0.49–2.28)	0.889
Constant			0.131 (0.03–0.59)	0.010
Number of observations			251	
Wald chi^2^ (14)			1596.77	
Prob > chi^2^			0.000	
Pseudo *R*^2^			0.1117	

a Significance at 5% level (corresponds to the multivariate results).

b Significance at 1% level (corresponds to the multivariate results).

TASAF, Tanzania Social Action Fund.

Distance to the healthcare facility was found to influence the likelihood of seeking healthcare services: households residing <5 km from a facility were 1.6 times more likely to seek care than those residing >5 km from the healthcare facility though not statistically significant (OR 1.62, 95% CI 0.74–5.44; *P* = 0.225). Lastly, household healthcare utilization was positively and significantly associated with continuous availability of all essential medicines for the surveyed facilities (OR 3.49, 95% CI 1.02–12.04; *P* = 0.047).

## Discussion

This study assessed medicine availability and stock-outs in public health facilities and examined the effects of medicines availability on healthcare utilization in six districts of Dodoma region in Tanzania. We found that the availability of most tracer medicines was relatively good, with continuous availability of ∼70% of the medicines assessed over a 3 months period, much higher compared with the findings in Malawi where overall availability of medicines in public facilities was <50% (Khuluza and Haefele-Abah, 2019). Frequent stock-outs (5/18) were found for a few medicines, such as amoxicillin syrup or cotrimoxazole suspension, paracetamol tabs and ferrous salt and folic acid. This trend varied across facility types and across the districts. Medicine stock-outs at facilities were frequently due to the failure of the health facility to plan for needed refills and to stock-outs at the central MSD.

Medicines such as paracetamol, ferrous salt and folic acid availability were low compared with the reported estimated in LMIC countries such as Nigeria (Sun *et al.*, 2018), Malawi (Khuluza and Haefele-Abah, 2019) and Ethiopia (Sado and Sufa, 2016). The reported causes for regular stock-outs at health facility level were related to procurement inefficiencies, staff ability to forecast needs and requisitioning of medical commodities (Walker and Ozawa, 2011). Therefore, improvements in communication, forecasting and ordering procedures at healthcare facilities are necessary for addressing such inefficiencies (Soyiri and Reidpath, 2013).

We found that the majority of households reported having sought care from public healthcare facilities, similarly to the findings of other studies (Basu *et al.*, 2012; Ngugi *et al.*, 2017). This finding shows the importance of the public sector in the provision of healthcare services, especially for the marginalized population. Among the prerequisites for UHC include ensuring availability of high-quality medicines in the public facilities, rational prescribing, strengthening the community and peripheral health facility level (WHO, 2012). The results of these studies indicated that the continued availability of essential medicines at the facility may influence the use of public health facility services.

The association between distance from a health facility and the use of health services was not statistically significant. Other recent studies found that living in the proximity (<1 h walking time) of a health facility increases the probability of household healthcare utilization(Buor, 2002; Anselmi *et al.*, 2015; Khuluza and Haefele-Abah, 2019), whereas in Vietnam those living <1 km were three times likely to utilize healthcare services compared with those residing >1 km from the facility (Tran *et al.*, 2016).

Waiting time was found to influence healthcare utilization as reported in other settings (Afolabi *et al.*, 2013; Sado and Sufa, 2016). In our analysis, we assessed waiting time as measured in terms of how long a client normally wait until s/he gets treatment contrary to that of Nigeria which was measured in terms of a four point’s Likert-scale (Afolabi *et al.*, 2013) and Laos which participants rated long clinic waiting time as one of the barriers in seeking treatment at the facilities (Phrasisombath *et al.*, 2012). Irrespective of the methodology used to assess the effect of waiting time on healthcare utilization, findings tend to be similar. In contrast, easy access, shorter waiting time and longer or flexible opening hours have been demonstrated to increase the use of formal healthcare services (Shaikh and Hatcher, 2005; Sado and Sufa, 2016).

We could not find an association between trust in healthcare providers and use of health services as it was found by other studies (Trachtenberg *et al.*, 2005; Dawson-Rose *et al.*, 2016). Trust in providers influences both healthcare-seeking, and influences patient engagement, participation in care and treatment adherence (Mkoka *et al.*, 2014). A high level of trust between the client and the provider induces people to utilize healthcare services from a given facility (Russell, 2005). Trust is defined as the household’s perceived technical competence of the healthcare provider (face-to-face interaction) (Russell, 2005; Dawson-Rose *et al.*, 2016) as well as inter-personal dimensions of quality of care (Russell, 2005). Stock-outs of medicines at the healthcare facility affects the quality of healthcare services which, in turn, undermine the trust which the population has in the health services influencing health-seeking behaviour (Mkoka *et al.*, 2014).

We found an association between health education and healthcare utilization from the study area. As documented elsewhere, health education impacts household knowledge and willingness to seek healthcare services from formal healthcare providers (Oladipo, 2014; Jibril *et al.*, 2017). Raising community awareness of health issues, illness prevention and encouragement of timely care-seeking, in turn, improve health outcomes.

Similar to the findings of other studies (Ahmed *et al.*, 2018; Atnafu *et al.*, 2018), we have found that CHF beneficiaries were more likely to seek healthcare in formal settings as compared with non-insured households. Financial protection is crucial in achieving UHC, implying the absence of (substantial) out-of-pocket payments when accessing healthcare services (Abiiro *et al.*, 2014; Ataguba and Ingabire, 2016). Insured households are less likely to delay care-seeking, borrow or sell their valuable assets or incur income loss when accessing care (Abiiro *et al.*, 2014). The government of Tanzania within its Health Sector Strategic Plan for 2015–2020 made commitments towards universal healthcare through social health insurance (URT, 2015). The health financing strategy includes the scale-up the coverage of redesigned CHFs (the so-called ‘CHF iliyoboreshwa’) with the aim of reaching all households. It is anticipated that the uptake of CHF iliyoboreshwa will improve household access to care as well as facility revenue. In turn, facilities could use the CHFs revenue, together with other cost-sharing mechanisms, to improve quality-of-care through procurement of medical commodities (medicines, medical equipment and medical supplies; Wiedenmayer *et al.*, 2019).

The results presented here should be considered alongside a few important limitations. First, we were unable to link data from many of the households with facility level data. This might lead to potential selection bias if the households we were able to link are systematically different from households we were unable to link. It could also influence the generalizability of the findings across the region. In addition, our study focused specifically on facilities in the public sector although households may seek care and services from the private sector too. The study focused only on the availability of medicines, as medical supplies and equipment data were limited. Lastly, respondents provided responses based on their past experiences and it is possible that responses were subject to some recall errors.

## Conclusion

This study showed that the availability of most tracer medicines was relatively good (compared with other studies in the region), although there were frequent stock-outs of a few medicines and wide variation across health facilities and district councils. Medicine availability was associated with higher use of healthcare services indicating it may play an important role in influencing household utilization of healthcare services in Tanzania. The results of this study highlight the importance of efficient co-ordination, planning and medicine supply management between the facility and the national supply chain. A better understanding of factors contributing to the performance of the Jazia PVS is crucial for improvement in medicines availability at the facilities. In addition, providers should consider the availability of healthcare services within a reasonable time as a way of shortening waiting time at the point of service. Moreover, healthcare providers should continue to provide healthcare education to the community in order to raise community awareness of relevant health issues and enhance knowledge in seeking timely and appropriate health assistance, along with community sensitization on the importance of health insurance in accessing healthcare services and avoiding health-related financial hardship.

## Supplementary Material

czz173_Supplementary_Data
